# Associations between maternal dietary scores during early pregnancy with placental outcomes

**DOI:** 10.3389/fnut.2023.1060709

**Published:** 2023-02-08

**Authors:** Shevaun M. Teo, Celine M. Murrin, John Mehegan, Alexander Douglas, James R. Hébert, Ricardo Segurado, Cecily C. Kelleher, Catherine M. Phillips

**Affiliations:** ^1^School of Public Health, Physiotherapy and Sports Science, University College Dublin, Dublin, Ireland; ^2^Cancer Prevention and Control Program and Department of Epidemiology and Biostatistics, Arnold School of Public Health, University of South Carolina, Columbia, SC, United States

**Keywords:** maternal diet quality, dietary inflammation, dietary scores, placental weight, placental development, birth weight

## Abstract

**Background and aims:**

Individual macronutrient and micronutrient effects on placental growth have been widely investigated. However, the influence of overall maternal diet is relatively unknown. Therefore, the aim of this study is to examine associations between a range of maternal dietary scores during early pregnancy with placental outcomes, and to investigate whether there is evidence of sexual dimorphism.

**Methods:**

This analysis of the Lifeways Cross-Generational Cohort includes 276 mother–child pairs. A validated 148-item semi-quantitative food frequency questionnaire assessed maternal diet in early pregnancy. Dietary scores reflecting dietary quality [Healthy Eating Index (HEI-2015), Dietary Approaches to Stop Hypertension (DASH)], dietary inflammatory potential [Dietary Inflammatory Index (DII) and the energy adjusted DII (E-DII)], dietary antioxidant status [Dietary Antioxidant Quality (DAQ)], and glycemic and insulinemic loads/indices (GL/GI, IL/II) were calculated. Linear regression analyses assessed maternal dietary score relationships with untrimmed placental weight (PW) and birth weight:placental weight (BW:PW) ratio.

**Results:**

In fully adjusted models, maternal E-DII and GI were positively associated, and HEI-2015 and DAQ were negatively associated with PW (B: 12.31, 95% CI: 0.41, 24.20, *p* = 0.04, B: 4.13, 95% CI: 0.10, 8.17, *p* = 0.04, B: −2.70, 95% CI: −5.03, −0.35, *p* = 0.02 and B: −15.03, 95% CI: −28.08, −1.98, *p* = 0.02, for E-DII, GI, HEI-2015 and DAQ respectively). Maternal DAQ associations with BW:PW ratio were attenuated. When stratified by sex, maternal GI and pregnancy-specific DAQ were associated with PW in female offspring (B: 5.61, 95% CI: 0.27, 10.96, *p* = 0.04 and B: −15.31, 95% CI: −30.35, −0.27, *p* = 0.046). Maternal E-DII and HEI-2015 were associated with PW in males (B: 24.31, 95% CI: 5.66, 42.96, *p* = 0.01 and B: −3.85, 95% CI: −7.47, −0.35, *p* = 0.03 respectively).

**Conclusion:**

The results of this novel investigation suggest that maternal diet may influence placental development. Female fetuses may be more sensitive to increased glucose levels whereas male fetuses may be more susceptible to *in-utero* stresses that are regulated by inflammatory pathways and overall diet quality. Hence, early pregnancy offers an opportune time for a mother to prioritize dietary changes that focus on reducing inflammatory and glycemic responses.

## 1. Introduction

The placenta is a key organ through which gasses, nutrients, and wastes are exchanged between maternal-fetal circulations (1). It undergoes adaptations in response to an adverse intrauterine environment to sustain its own adequate function and fetal survival (2). The Barker hypothesis proposes that the origins of chronic diseases in adulthood stem from these alterations in the supply of nutrients to the fetus through the placenta (3). Placental weight (PW) is a powerful independent predictor of birth weight (BW) (4, 5), and the BW:PW ratio is considered a marker of placental efficiency (6). There is growing interest in examining the influence of the early-life dietary environment (using holistic approaches such as dietary scores) on birth outcomes. A recent individual participant data (IPD) meta-analysis consisting of 24,861 mother–child pairs across seven European birth cohorts has provided robust evidence that a low quality and more pro-inflammatory maternal diet, characterized by lower Dietary Approaches to Stop Hypertension (DASH) and higher energy-adjusted Dietary Inflammatory Index (E-DII) scores, is associated with low birth weight (LBW) and higher risk of offspring being born small-for-gestational age (7, 8). However, examination of these maternal dietary scores in the context of placental outcomes is limited.

Reduced BW:PW ratios observed in infants with fetal growth restriction (FGR) may be indicative of inadequate placental nutrient transfer, failure to adapt according to fetal nutrient demand, and may confer increased chronic disease risk (6). Higher BW:PW ratio indicates increased placental efficiency and implies that nutrient transfer per gram placenta has increased to maintain fetal development and survival (6, 9). The greatest differences in BW:PW ratios have been observed at earlier gestational ages (10), therefore the influence of gestational age on placental outcomes also should be considered. Male infants are generally heavier and longer than females (11–13), with many studies reporting significant differences in PW between sexes (14–16). A dominant concept in the literature is that female and male fetuses have varying growth strategies in response to maternal diet (17), and hence, different offspring outcomes (8, 15, 18). While the mechanisms underlying these sex-related differences are not clearly understood, placental epigenetics may play an important role (19). However, there are no comparative studies conducted thus far which have examined the relationship between maternal dietary scores and placental outcomes in humans. In the one study available, secondary analysis of the PEARS RCT (*n* = 434) showed no association between the E-DII score and placental weight (20).

The aim of this study was to therefore investigate associations of maternal diet in early pregnancy, using a variety of validated dietary scores reflective of dietary quality [Healthy Eating Index (HEI-2015), DASH scores], dietary inflammatory potential [Dietary Inflammatory Index (DII) and the E-DII], dietary antioxidant status [Dietary Antioxidant Quality (DAQ) scores], and glycemic and insulinemic loads/indices (GL/GI, IL/II) with PW and BW:PW ratio. In addition, the study aimed to explore the effect of sexual dimorphism and gestational age on maternal diet-placental outcome associations.

## 2. Material and methods

### 2.1. Study population and setting

The Lifeways Cross-Generation Cohort Study is a prospective family study which has been described in detail elsewhere (21, 22). The study objectives were to document health status, diet and lifestyle in the family members and to establish patterns and links across generations. In brief, a cohort of 1094 infants were born to 1082 recruited mothers with questionnaire data between 2001-2003 in two national maternity hospitals in the Republic of Ireland, including 12 sets of twins. Of the mothers with singleton births (*n* = 1,114), 286 mothers had data on maternal diet and placental and birth outcomes available. The large majority (276/286) of placental weight data in the study came from the Galway cohort recruited at University College Hospital, Galway (UCHG) where placental data was routinely collected using the Euroking standardized maternity information system (Wellbeing Software, Mansfield, United Kingdom). This system was not used at the Coombe University Hospital, Dublin where two thirds of infants were born. Due to hospital catchment differences and to maintain a more homogenous sample, the 10 mothers from Dublin with placental data were excluded, resulting in a core study sample of 276 mother–child pairs for the current analysis. The participant flow chart is shown in Supplementary Data File. Ethical approval was granted by the ethics committees of the Coombe University Hospital, Dublin, University College Dublin, Irish College of General Practitioners and University College Hospital, Galway, Ireland. Written informed consent was collected from all women upon recruitment.

### 2.2. Dietary assessment

At recruitment during their first antenatal visit (between 12 and 16 weeks of pregnancy), mothers were given a questionnaire with sections relating to general health, smoking and alcohol use, and social characteristics to complete at home and return by mail. Diet was assessed using a modified version of the self-completed European Prospective Investigation into Cancer and Nutrition (EPIC) FFQ (23), which has been validated extensively in several populations (24). Adapted to reflect the Irish diet, the 148-item semi quantitative FFQ used in Lifeways was originally validated for use in the Irish population using food diaries and a protein biomarker in a volunteer sample (25) and incorporated into the Irish National Surveys of Lifestyle Attitudes and Nutrition 1998, 2002, 2007 (26–28). The FFQ was also validated using a 7-day weighed food record completed by a subsample of the Lifeways at 5-year follow-up, with reasonable agreement (*r* > 0.40) for fat, carbohydrate, and their components, and with lower agreement for protein (*r* = 0.25) (29). Participants were asked about their average consumption frequency (nine levels, from “never or less than once per month” to “6+ per day”) of each food item since becoming pregnant. The daily quantities of food intakes were then derived by multiplying the frequencies per day by the standard portion sizes. The FFQ also assessed the use of dietary supplements.

### 2.3. Nutrient and energy intake

Daily energy and nutrient intake was computed using an in-house software program (FFQ Software Ver 1.0©; developed by the National Nutrition Surveillance Center, School of Public Health, Physiotherapy and Sports Science, University College Dublin), which linked frequency selections with the food equivalents in McCance and Widdowson Food Tables (30). This software uses standard food portion sizes for each food item and uses the food frequency data to estimate intakes of the food in grams per day. Standard food composition data are then applied (frequency weight × nutrient content) to estimate the nutrient intake per day. Implausible energy intakes (<500 kcal/day or >3,500 kcal/day) were excluded from the analysis (31).

### 2.4. Dietary scores

#### 2.4.1. Healthy eating Index-2015 (HEI-2015)

The HEI-2015 is a measure of overall diet quality that measures alignment with the 2015–2020 American dietary guidelines (32). The HEI-2015 contains 13 components which are scored on a density basis out of 1,000 calories, with the exception of fatty acids, which are a ratio of unsaturated to saturated fatty acids (SFAs) (33). As the HEI is originally a scoring system developed in the United States, the USDA has developed the Food Patterns Equivalent Database (FPED) to provide the HEI component cup/oz. equivalent per 100 g for a large range of food items. Each of the foods used in the FFQ in the present study was matched to a corresponding food in the FPED, and these HEI cup/oz. equivalents then used in the calculation of the HEI scores. The entire calculation is done with these cup/oz. equivalents, so there is no need to convert from cups/oz. to grams. Total fruits, whole fruits, total vegetables, greens and beans, total protein containing foods, and seafood and plant proteins scored 5 in the highest consumption and 0 in the lowest consumption. The highest consumption of three components including whole grains, dairy, and fatty acids (ratio of poly- and monounsaturated fatty acids (PUFAs and MUFAs) to SFAs) scored as 10 and the lowest consumption scored as 0. Four components (refined grains, sodium, added sugars, and saturated fats) scored 10 in the lowest consumption and 0 in the highest consumption (33). As previously described (34), component scores were summed to yield a total score ranging from 0 to 100, with a higher score indicating higher diet quality.

#### 2.4.2. Dietary approaches to stop hypertension (DASH) score

Diet quality was also assessed by adherence to the DASH diet, which has been promoted by the US National Heart, Lung and Blood Institute to prevent and control hypertension. DASH score generation has been described elsewhere (8, 35). In brief, in the Lifeways Study, 85 FFQ food items (excluding alcohol) were used to create the DASH score based on the index proposed by Fung et al. (36). This scoring method was based on the quintile distribution (i.e., ranking) of energy adjusted intakes of the following components: high consumption of fruits, vegetables, nuts, seeds and legumes, low-fat dairy products and whole grains, and a low consumption of sodium, sweetened beverages, sweets and added sugars, and red and processed meats (37). The overall DASH scores ranged from 8 to 40, with a higher DASH score reflecting a higher diet quality. Two DASH scores were created; a DASH score based on frequency of intake (e.g., “once a week” was converted to daily frequency using the formula 1/7) and a DASH score based on amounts (measured in grams/day).

#### 2.4.3. Dietary inflammatory index (DII and E-DII)

Maternal dietary inflammatory potential was determined using the DII and its energy-adjusted counterpart, the E-DII. These are validated, literature-derived scores based on the original DII, whose calculation has been described in detail previously (38). In brief, the DII combines a range of macronutrients and micronutrients with various non-nutrient naturally occurring compounds in food (e.g., caffeine and flavanols) and herbs and spices (such as onion, turmeric, saffron, thyme, rosemary, and green and black tea) (38). The score represents the overall inflammatory potential of an individual’s diet based on up to 45 pro- and anti-inflammatory food parameters that either increased or decreased six circulating biomarkers of inflammation (CRP, IL1β, IL4, IL6, IL10, and TNFα). Full details on DII determination in the Lifeways cohort have been published previously (39, 40). A total of 28 of the 45 possible food parameters were used for DII calculation based on the FFQ in this study and these were as follows: carbohydrate, protein, fat, alcohol, fiber, cholesterol, saturated fat, mono-unsaturated fat, poly-unsaturated fat, niacin, thiamin, riboflavin, vitamin B12, vitamin B6, iron, magnesium, zinc, selenium, beta-carotene, vitamin A, vitamin C, vitamin D, vitamin E, folic acid, onion, garlic, tea, and caffeine. For the E-DII, energy was in the denominator; so, 27 parameters were used for computation. For both the DII and E-DII, higher scores are more pro-inflammatory and lower scores are more anti-inflammatory. The DII and E-DII are scored similarly and scaled identically, hence the scores are comparable across studies (41).

#### 2.4.4. Dietary antioxidant quality (DAQ) score

The DAQ Score summarizes certain vitamins and minerals that have been proven to act as dietary antioxidants (selenium, zinc, vitamin A, vitamin C, and vitamin E). It sums and assigns a score relative to the US Food and Drug Administration recommended quantity and was developed to determine the overall effect of antioxidants on health outcomes. The Tur et al. (42) method was used in the calculation of the daily nutrient intake of these micronutrients. This score was modified for use in a pregnant Irish population using the European Food Safety Authority and Food Safety Authority Ireland dietary reference values (DRV), namely the Irish recommended dietary allowance (RDA) values. Two DAQ scores were generated; one based on the Irish RDAs for the general female population (>18 years) and one based on the Irish RDAs during pregnancy. Intakes below 2/3 of the RDA was the criterion used to estimate the risk of inadequate intake of each of the 5 antioxidant nutrients and was scored as 0; intakes higher than 2/3 of the RDA were scored as 1. Thus, the total dietary antioxidant quality score ranged from 0 (very poor quality) to 5 (high quality).

#### 2.4.5. Glycemic index (GI) and glycemic load (GL)

The GI and GL have been proposed as measures that qualify and quantify the postprandial glycaemic responses according to the dietary carbohydrate intake. Calculation of dietary GI and GL in the Lifeways cohort has been described in full detail elsewhere (43). The GL for individual food items was calculated by multiplying the amount of available carbohydrate in one serving of food item by its GI value, then dividing by 100 (44). Average participant GL was then derived by summing the food intake frequency-weighted GL for all food items. The average dietary GI of participants was calculated by dividing their GL by their total available carbohydrate intake, then multiplying by 100.

#### 2.4.6. Insulinemic index (II) and insulinemic load (IL)

The dietary insulinemic index (II) and its corresponding insulinemic load (IL) are measures of insulin demand elicited by various foods. For the Lifeways cohort, II values were assigned to each food recorded in the FFQ according to a standardized procedure (45). Based on these II values, average dietary IL for participants was calculated by multiplying the II value of each food by its energy content and the consumption frequency and summing over all reported food items (43, 45). The average dietary II was calculated by dividing the average IL by the total energy intake.

### 2.5. Placental and birth outcomes

As part of standard obstetric practice, the umbilical cord was clamped post-delivery and the placenta weighed within the delivery room (i.e., the untrimmed weight). This weight was then recorded using the Euroking maternity information system (Wellbeing Software, Mansfield, United Kingdom).

Birth weight and birth length and infant sex were abstracted from linked hospital records. BW:PW ratio was calculated by dividing the birth weight by the untrimmed placental weight. Ponderal index (PI) was calculated using the formula weight (kg)/length (m)^3^.

### 2.6. Covariates

At recruitment, mothers provided information on age, self-reported height, pre-pregnancy weight, marital status, socioeconomic status [proxied by eligibility to the General Medical Services (GMS) Scheme, a robust indicator of social disadvantage in Ireland] and highest education attainment (tertiary or no tertiary education) using a self-completion questionnaire. Pre-pregnancy BMI was subsequently derived using the formula weight (kg)/height (m)^2^. Physical activity levels, alcohol intake and cigarette smoking during pregnancy also were ascertained using the same questionnaire (current smokers/drinkers and women who smoked/consumed alcohol less than 3 months prior to recruitment were classified as “exposed”) (34).

### 2.7. Statistical analysis

Summary descriptive statistics were generated for maternal and child characteristics. The normality of distribution for each of the outcome measures was examined visually using the frequency distribution (histogram) and Q-Q plot (quantile-quantile plot). No formal test of normality (e.g., the Shapiro–Wilk test) on the outcome data was applied, as a significant test result does not necessarily invalidate the methods applied. Visual inspection of the residuals from regression analysis was performed (histograms and Q-Q plots), to verify this assumption. Under the Central Limit Theorem, the means and parameter estimates from linear regression will be normally distributed with a large sample size.

Differences in characteristics between the Galway cohort (*n* = 276) included in the current analysis and all other Lifeways participants (*n* = 662) were assessed using one way-ANOVA for continuous variables and the chi-square test for categorical variables. Associations between maternal dietary scores and PW, BW:PW ratio and BW were examined using linear regression analyses. The main outcome measures were PW and BW:PW ratio, with BW being a secondary outcome of interest. Three models were run: model 1 was an unadjusted model, model 2 adjusted for peripregnancy variables (maternal age, maternal pre-pregnancy BMI, maternal height, energy intake, gestational age, offspring sex and parity), and model 3 additionally adjusted for maternal education and lifestyle factors (smoking, physical activity and alcohol intake). Due to inherent energy adjustment, it was not necessary to adjust for energy intake for the E-DII score and the DASH scores. Linear regression analyses were repeated stratifying by sex to examine sex differences. As extreme outliers, defined as values greater than 3 standard deviations away from the mean (46), were identified for the GI/GL/II/IL scores, sensitivity-to-outliers regression was conducted to assess whether the associations between glycemic and insulinemic scores with PW, BW:PW and BW were sensitive to outliers (*n* = 13) by excluding them from these analyses. Ordered leverage values and Cook’s Distance values were also inspected for the Galway cohort (*n* = 276) to determine whether any of the identified outlier cases were highly influential points. Sensitivity analyses were also conducted to assess robustness of overall results when restricted to term infants by excluding infants born pre-term (<37 weeks) and post-term (>42 weeks). When restricting to term births, mothers without gestational age (*n* = 12) were excluded from analyses. To correct for the multiple testing performed and control Type I errors, false discovery rate (FDR)-adjusted *p* values were calculated using the method described by Benjamini and Hochberg (46). Results presented refer to the non-FDR-adjusted two-sided *p* value of <0.05 as being considered statistically significant. Statistical analyses were conducted using the statistical software SPSS version 26.0 (IBM Corporation, Armonk, NY, United States).

## 3. Results

### 3.1. Characteristics of the study population mothers

The characteristics of the mothers included in this analysis (*n* = 276) are presented in Table 1. Overall, the mothers had a mean age of 31.9 ± 5.2 years and mean BMI of 23.8 ± 4.0 kg/m^2^. Most mothers (64%) had a tertiary level of education and had previously given birth (61%). Regarding lifestyle factors, 17% reported smoking, 68% drank alcohol and 21% engaged in regular physical activity during the periconceptional period. None of the mothers were diagnosed with gestational diabetes (GDM). Comparison of these mothers with the rest of the Lifeways cohort (*n* = 662) revealed some differences in maternal age, height, education level, marital status, smoking status during pregnancy, gestational age and placental weight in keeping with their hospital catchment areas (*p* < 0.05).

**Table 1:** Characteristics of the Lifeways study mothers.

Maternal characteristics	All Lifeways mothers	Galway mothers with placental data	Rest of the Lifeways cohort	*p*-value^1^
Age at recruitment (years)	30.36 (5.77)	31.89 (5.17)	29.72 (5.89)	**<0.001** ^2^
Pre-pregnancy BMI (kg/m^2^)	23.89 (4.18)	23.79 (4.04)	23.94 (4.25)	0.65
Weight (kg)	63.8 (11.23)	64.57 (10.92)	63.45 (11.36)	0.19
Height (cm)	163.75 (6.42)	164.68 (6.09)	163.31 (6.54)	**0.004**
Parity (non-nulliparous)	642 (56.1%)	169 (61.2%)	362 (54.7%)	0.07
Education level				**<0.001**
Below tertiary	455 (49.5%)	100 (36.4%)	355 (55%)
Tertiary or above	465 (50.5%)	175 (63.6%)	290 (45%)
Smoking during pregnancy	234 (24.9%)	46 (16.7%)	188 (28.4%)	**<0.001**
Alcohol use during pregnancy	652 (69.9%)	187 (67.8%)	465 (70.8%)	0.36
Regular physical activity	162 (19.5%)	56 (21%)	106 (18.8%)	0.47
Maternal energy intake (kcal/day)	2223.31 (588.57)	2211.12 (565)	2228.39 (598.47)	0.68
Marital status				**<0.001**
Married/cohabiting	737 (79.2%)	250 (90.9%)	487 (74.4%)
Separated/divorced/single	193 (20.8%)	25 (9.1%)	168 (25.6%)
Household weekly income				0.12
<€200	62 (7.4%)	19 (7.4%)	43 (7.4%)
€200–€600	472 (56.2%)	158 (61.2%)	314 (54%)
>€600	306 (36.4%)	81 (31.4%)	225 (38.7%)	
Eligibility for GMS^3^	153 (16.3%)	38 (14%)	114 (17.3%)	0.21
Private health insurance	446 (49%)	178 (65.4%)	268 (42.0%)	**<0.001**

Values are presented as mean (SD) for continuous variables or *n* (%) for categorical variables.

1 *p*-value refers to statistical tests between Galway mothers with placental data (*n* = 276) and the rest of the Lifeways cohort (*n* = 662). Significant *p*-values are highlighted in bold.

2 Welch statistic used due to not meeting the assumption of homogeneity of variances.

3 GMS, General Medical Services.

### 3.2. Offspring characteristics

Characteristics of the offspring with placental data (*n* = 276, 48.9% male), full sample and stratified by sex and those without placental data (*n* = 662, male 51.4%) are shown in Table 2. The mean birthweight in this study was 3575.33 ± 513.49 g and the mean placental weight was 725.25 ± 143.76 g. Significant differences between sexes were observed for head circumference (*p* = 0.003). No significant differences were observed between the sexes for the three main outcomes of interest. Differences between the Galway cohort, included in this study, and the rest of the Lifeways cohort were observed for gestational age, head circumference and placental weight (*p* < 0.05).

**Table 2:** Galway cohort maternal dietary scores and offspring characteristics, overall and stratified by sex.

Maternal dietary score	Galway mothers with placental data	Rest of the Lifeways cohort	*p*-value^1^	Galway male offspring	Galway female offspring	*p*-value^2^
DII^1^	−0.29 (1.70)	0.23 (1.77)	**<0.01**	−0.28 (1.76)	−0.29 (1.64)	0.99
E-DII^1^	−0.19 (1.60)	0.43 (1.67)	**<0.01**	−0.28 (1.76)	−0.24 (1.55)	0.58
HEI-2015^2^	53.72 (8.42)	51.16 (8.33)	**<0.01**	53.36 (8.90)	54.07 (7.94)	0.48
DASH (frequencies)^2^	24.68 (4.79)	23.90 (4.84)	**0.02**	24.17 (4.82)	25.18 (4.72)	0.08
DASH (weight)^2^	24.88 (5.00)	23.87 (5.05)	**<0.01**	24.34 (5.02)	25.40 (4.94)	0.08
DAQ General^2^	3.51 (1.49)	3.57 (1.39)	0.53	3.44 (1.55)	3.57 (1.44)	0.47
DAQ Pregnancy^2^	3.36 (1.50)	3.41 (1.42)	0.64	3.28 (1.53)	3.44 (1.48)	0.40
GI^1^	59.42 (4.54)	58.59 (4.91)	**0.02**	59.42 (4.96)	59.43 (4.12)	0.98
GL^1^	153.31 (49.28)	150.70 (49.34)	0.46	152.60 (50.16)	154.00 (48.59)	0.81
II^1^	57.06 (15.75)	57.76 (13.56)	0.50	56.20 (16.25)	57.89 (15.27)	0.37
IL^1^	704.48 (273.10)	648.25 (261.06)	**<0.01**	710.78 (307.23)	698.46 (236.82)	0.71^3^
*Offspring characteristic*						
Child sex			0.50	48.9%	51.1%	N/A
Female	141 (51.1%)	322 (48.6%)
Male	135 (48.9%)	340 (51.4%)
Gestational age (weeks)	39.84 (1.58)	40.09 (1.84)	**0.05**	39.85 (1.68)	39.82 (1.49)	0.89
Placental weight (g)	725.25 (143.76)	609.1 (123.02)	**0.01**	731.81 (166.60)	718.97 (118.10)	0.46^3^
BW:PW ratio	5.02 (0.68)	4.61 (1.60)	0.44^3^	5.02 (0.70)	5.02 (0.67)	0.99
Birth weight (g)	3575.33 (513.49)	3505.10 (577.14)	0.07^3^	3602.34 (603.64)	3549.29 (408.76)	0.39
Birth length (cm)	50.75 (2.30)	50.63 (3.06)	0.55^3^	50.99 (2.64)	50.51 (1.91)	0.30^3^
Head circumference (cm)	35.13 (1.40)	34.90 (1.92)	**0.05** ^3^	35.39 (1.49)	34.89 (1.27)	**0.003**
Ponderal index (kg/m^3^)	27.26 (2.56)	26.89 (5.12)	0.17^3^	27.01 (2.85)	27.51 (2.22)	0.11^3^

Values are presented as mean (SD) for continuous variables or *n* (%) for categorical variables.

1 *p*-value refers to statistical tests between Galway cohort (*n* = 276) and the rest of the Lifeways cohort (*n* = 662).

2 *p*-value refers to statistical tests between male offspring (*n* = 135) and female offspring characteristics (*n* = 141).

3 Welch statistic used due to not meeting the assumption of homogeneity of variances. Significant *p*-values are highlighted in bold.

### 3.3. Maternal dietary score associations with placental weight, BW:PW ratio, and birth weight

#### 3.3.1. Maternal dietary scores and placental weight

The results of the linear regression analyses examining maternal diet associations with placental weight are shown in Table 3. Maternal GI was positively associated with PW in the crude model and persisted in the fully adjusted model, where a one-unit increase in the GI score was associated with an increase in PW of 4.13 g. Negative associations between the general and pregnancy-specific DAQ scores and PW remained significant in fully adjusted models (B: −15.03, *p* = 0.02 and B: −13.15, *p* = 0.03, respectively), indicating that a higher maternal dietary antioxidant status is associated with lower placental weights. For E-DII and HEI-2015 scores, associations were observed only after final adjustment for all covariates. Higher HEI-2015 scores (reflecting better diet quality) were associated with lower placental weights (B: −2.69, *p* = 0.02) while higher E-DII, but not DII, scores indicative of a more pro-inflammatory diet were associated with higher placental weights (B: 12.31, *p* = 0.04).

**Table 3:** Associations between maternal dietary scores and placental weight (g) in the full sample and stratified by sex.

	Model 1 unadjusted			Model 2			Model 3		
	B	95% CI	*p*-value	B	95% CI	*p*-Value	B	95% CI	*p*-value
**All Offspring**									
DII^1^	1.12	(−2.63, 25.19)	0.83	8.59	(−4.99, 22.16)	0.21	11.28	(−2.63, 25.19)	0.11
E-DII^1^	7.58	(−3.04, 18.20)	0.16	9.42	(−2.06, 20.90)	0.11	12.31	(0.41, 24.20)	**0.04**
HEI-2015^2^	−1.84	(−3.86, 0.18)	0.07	−2.08	(−4.32, 0.17)	0.07	−2.69	(−5.03, −0.35)	**0.02**
DASH (frequencies)^2^	−2.23	(−5.79, 1.33)	0.22	−1.47	(−5.40, 2.45)	0.46	−2.79	(−7.00, 1.41)	0.19
DASH (weight)^2^	−2.46	(−5.87, 0.95)	0.16	−1.97	(−5.74, 1.80)	0.30	−3.55	(−7.67, 0.56)	0.09
DAQ General^2^	−10.94	(−22.38, 0.50)	0.06	−12.86	(−24.85, −0.88)	**0.04**	−15.03	(−28.08, −1.98)	**0.02**
DAQ Pregnancy^2^	−9.88	(−21.25, 1.49)	0.09	−11.98	(−23.91, −0.05)	**0.05**	−13.16	(−25.19, −1.12)	**0.03**
GI^1^	4.50	(0.77, 8.22)	**0.02**	3.51	(−0.38, 7.40)	0.08	4.13	(0.10, 8.17)	**0.04**
GL^1^	0.23	(−0.11, 0.58)	0.19	0.21	(−0.14, 0.56)	0.23	0.21	(−0.15, 0.57)	0.25
II^1^	−0.54	(−1.63, 0.54)	0.33	−0.38	(−1.51, 0.74)	0.50	−0.40	(−1.54, 0.75)	0.49
IL^1^	0.08	(0.02, 0.14)	**0.01**	0.07	(0.002, 0.13)	**0.04**	0.06	(−0.01, 0.12)	0.10
**Males**									
DII^1^	6.75	(−9.45, 22.96)	0.41	22.12	(0.52, 43.72)	**0.04**	23.09	(1.44, 44.74)	**0.04**
E-DII^1^	19.24	(2.38, 36.09)	**0.03**	21.46	(3.35, 39.56)	**0.02**	24.31	(5.66, 42.96)	**0.01**
HEI-2015^2^	−3.31	(−6.47, −0.15)	**0.04**	−3.85	(−7.35, 0.35)	**0.03**	−3.85	(−7.47, −0.24)	**0.04**
DASH (frequencies)^2^	−3.36	(−9.26, 2.54)	0.26	−2.47	(−9.07, 4.14)	0.46	−1.07	(−8.64, 6.50)	0.78
DASH (weight)^2^	−4.20	(−9.85, 1.44)	0.14	−3.79	(−10.14, 2.56)	0.24	−2.89	(−10.42, 4.64)	0.45
DAQ General^2^	−8.85	(−27.31, 9.61)	0.34	−10.82	(−30.15, 8.51)	0.27	−14.56	(−33.22, 4.10)	0.12
DAQ Pregnancy^2^	−7.06	(−25.77, 11.65)	0.46	−9.82	(−29.33, 9.70)	0.32	−14.21	(−33.03, 4.61)	0.14
GI^1^	3.60	(−2.13, 9.33)	0.22	2.68	(−3.18, 8.54)	0.37	3.24	(−2.92, 9.40)	0.30
GL^1^	0.26	(−0.30, 0.83)	0.36	0.29	(−0.28, 0.85)	0.32	0.24	(−0.36, 0.84)	0.43
II^1^	−0.87	(−2.62, 0.89)	0.33	−0.68	(−2.45, 1.09)	0.45	−0.75	(−2.55, 1.05)	0.41
IL^1^	0.07	(−0.02, 0.16)	0.13	0.07	(−0.03, 0.16)	0.15	0.05	(−0.05, 0.15)	0.33
**Females**									
DII^1^	−5.07	(−17.10, 6.96)	0.41	−4.93	(−21.39, 11.53)	0.55	−4.58	(−22.18, 13.02)	0.61
E-DII^1^	−5.55	(−18.29, 7.21)	0.39	−4.31	(−18.13, 9.51)	0.54	−3.67	(−18.89, 11.54)	0.63
HEI-2015^2^	−0.01	(−2.50, 2.48)	0.99	0.56	(−2.28, 3.40)	0.70	0.06	(−3.00, 3.12)	0.97
DASH (frequencies)^2^	−0.88	(−5.07, 3.31)	0.68	−0.22	(−4.86, 4.42)	0.93	−0.82	(−5.80, 4.15)	0.74
DASH (weight)^2^	−0.52	(−4.52, 3.49)	0.80	0.05	(−4.38, 4.49)	0.98	−0.84	(−5.73, 4.06)	0.74
DAQ General^2^	−12.93	(−26.61, 0.75)	0.06	−13.25	(−27.83, 1.32)	0.07	−15.16	(−30.49, 0.16)	0.052
DAQ Pregnancy^2^	−12.41	(−25.72, 0.91)	0.07	−12.61	(−26.81, 1.60)	0.08	−15.31	(−30.35, −0.27)	**0.046**
GI^1^	5.74	(1.03, 10.46)	**0.02**	5.12	(0.03, 10.21)	**0.05**	5.61	(0.27, 10.96)	**0.04**
GL^1^	0.21	(−0.20, 0.61)	0.32	0.20	(−0.22, 0.62)	0.35	0.19	(−0.24, 0.63)	0.38
II^1^	−0.15	(−1.44, 1.15)	0.82	0.27	(−1.13, 1.67)	0.70	0.27	(−1.17, 1.71)	0.71
IL^1^	0.09	(0.01, 0.17)	**0.03**	0.07	(−0.01, 0.16)	0.09	0.07	(−0.02, 0.16)	0.11

B, unstandardized regression coefficient; CI, Confidence interval. *n* = 276 for all offspring; *n* = 135 for male offspring; *n* = 141 for female offspring. Model 1 = crude unadjusted model; Model 2 adjusts for: maternal age at recruitment, maternal pre-pregnancy BMI, length of gestation, offspring sex, parity and maternal height. Final model adjusted for: Model 2 plus smoking, physical activity, alcohol intake, energy intake (when not already considered in dietary score/%EI calculation), maternal education. E-DII, energy-adjusted Dietary Inflammatory Index; DASH, Dietary Approaches to Stop Hypertension; DAQ, Dietary Antioxidant Quality score; HEI, Healthy Eating Index; GI, glycaemic index; GL, glycaemic load; II, insulinemic index; IL, insulinemic load.Significant *p*-values are highlighted in bold.

1 Higher scores indicate less favorable dietary score.

2 Higher scores indicate more favorable dietary score.

In sex-stratified analyses maternal DII, E-DII and HEI-2015 were associated with PW in males (B: 23.1, *p* = 0.04; B: 24.3, *p* = 0.01; and B: −3.86, *p* = 0.04 respectively) in fully adjusted models. In female offspring, maternal GI was persistently positively associated with PW, with a larger magnitude of effect compared to the male offspring (B: 5.61, *p* = 0.04 vs. B:3.24, *p* = 0.30 respectively). Both DAQ scores were also negatively associated with PW in female offspring (*p* = 0.05), suggesting that improved maternal dietary antioxidant status is associated with lower placental weights in female offspring. None of the above findings persisted after FDR adjustment.

#### 3.3.2. Maternal dietary scores and BW:PW ratio

The results of linear regression analyses examining maternal diet associations with BW:PW ratio are shown in Table 4. Positive associations between both DAQ scores were found in the crude unadjusted model only, suggesting that improved antioxidant status is associated with improved placental efficiency (*p* < 0.05). However, no significant associations were found between any of the maternal dietary scores and BW:PW ratio following adjustment for potential confounders. There were also no associations between any of the maternal dietary scores and BW:PW ratio in either sex. It is important to note that these models had a very small adjusted *R^2^* value and did not predict BW:PW ratio to a statistically significant level based on the ANOVA table (47).

**Table 4:** Associations between maternal dietary scores and Birth weight:placental weight (BW:PW) ratio in the full sample and stratified by sex.

	Model 1 unadjusted			Model 2			Model 3		
	Bs	95% CI	*p*-value	B	95% CI	*p*-value	B	95% CI	*p*-value
**All offspring**									
DII^1^	−0.001	(−0.050, 0.048)	0.97	−0.015	(−0.082, 0.051)	0.65	−0.019	(−0.088, 0.050)	0.59
E-DII^1^	−0.026	(−0.078, 0.026)	0.33	−0.017	(−0.074, 0.039)	0.55	−0.021	(−0.080, 0.038)	0.48
HEI-2015^2^	0.008	(−0.002, 0.018)	0.10	0.009	(−0.002, 0.020)	0.10	0.011	(−0.001, 0.022)	0.07
DASH (frequencies)^2^	0.006	(−0.012, 0.023)	0.53	0.006	(−0.014, 0.025)	0.57	0.008	(−0.013, 0.028)	0.47
DASH (weight)^2^	0.005	(−0.012, 0.022)	0.56	0.005	(−0.014, 0.023)	0.63	0.007	(−0.013, 0.028)	0.49
DAQ General^2^	0.061	(0.007, 0.115)	**0.03**	0.049	(−0.010, 0.108)	0.10	0.051	(−0.009, 0.111)	0.10
DAQ Pregnancy^2^	0.059	(0.005, 0.113)	**0.03**	0.046	(−0.012, 0.105)	0.12	0.048	(−0.011, 0.108)	0.11
GI^1^	−0.004	(−0.022, 0.014)	0.67	−0.005	(−0.024, 0.014)	0.60	−0.006	(−0.025, 0.014)	0.58
GL^1^	−0.001	(−0.003, 0.001)	0.25	−0.001	(−0.003, 0.001)	0.26	−0.001	(−0.003, 0.001)	0.26
II^1^	−0.000063	(−0.005, 0.005)	0.98	0.001	(−0.005, 0.006)	0.79	0.001	(−0.005, 0.006)	0.83
IL^1^	−0.0002	(−0.0005, 0.0001)	0.21	−0.0002	(−0.0006, 0.0001)	0.12	−0.0003	(−0.0006, 0.0001)	0.12
**Males**									
DII^1^	−0.025	(−0.09, 0.04)	0.47	−0.036	(−0.13, 0.06)	0.46	−0.035	(−0.14, 0.07)	0.49
E-DII^1^	−0.061	(−0.13, 0.01)	0.09	−0.036	(−0.12, 0.05)	0.38	−0.036	(−0.12, 0.05)	0.40
HEI-2015^2^	0.013	(0.00, 0.03)	0.06	0.011	(0.00, 0.03)	0.15	0.014	(0.00, 0.03)	0.10
DASH (frequencies)^2^	0.006	(−0.02, 0.03)	0.66	−0.002	(−0.03, 0.03)	0.87	−0.001	(−0.03, 0.03)	0.96
DASH (weight)^2^	0.007	(−0.02, 0.03)	0.56	−0.001	(−0.03, 0.03)	0.92	−0.001	(−0.03, 0.03)	0.95
DAQ General^2^	0.045	(−0.03, 0.12)	0.25	0.034	(−0.05, 0.12)	0.43	0.040	(−0.05, 0.13)	0.36
DAQ Pregnancy^2^	0.033	(−0.04, 0.11)	0.40	0.026	(−0.06, 0.11)	0.54	0.031	(−0.06, 0.12)	0.48
GI^1^	0.008	(−0.016, 0.032)	0.49	0.011	(−0.014, 0.037)	0.38	0.011	(−0.016, 0.038)	0.43
GL^1^	−0.001	(−0.003, 0.002)	0.60	−0.001	(−0.003, 0.002)	0.63	−0.0005	(−0.003, 0.002)	0.72
II^1^	0.002	(−0.006, 0.009)	0.69	0.004	(−0.004, 0.011)	0.34	0.004	(−0.004, 0.012)	0.35
IL^1^	−0.0001	(−0.0005, 0.0003)	0.53	−0.0002	(−0.0006, 0.0002)	0.30	−0.0002	(−0.0006, 0.0003)	0.41
**Females**									
DII^1^	0.006	(−0.06, 0.07)	0.86	0.006	(−0.09, 0.10)	0.90	0.014	(−0.08, 0.11)	0.77
E-DII^1^	0.011	(−0.06, 0.08)	0.76	0.009	(−0.07, 0.09)	0.83	0.013	(−0.07, 0.10)	0.75
HEI-2015^2^	0.004	(−0.01, 0.02)	0.58	0.004	(−0.01, 0.02)	0.62	0.006	(−0.01, 0.02)	0.49
DASH (frequencies)^2^	0.009	(−0.01, 0.03)	0.44	0.010	(−0.02, 0.04)	0.44	0.011	(−0.02, 0.04)	0.44
DASH (weight)^2^	0.005	(−0.02, 0.03)	0.64	0.007	(−0.02, 0.03)	0.57	0.008	(−0.02, 0.04)	0.54
DAQ General^2^	0.063	(−0.01, 0.14)	0.11	0.052	(−0.03, 0.14)	0.23	0.071	(−0.01, 0.16)	0.10
DAQ Pregnancy^2^	0.066	(−0.01, 0.14)	0.08	0.055	(−0.03, 0.14)	0.19	0.075	(−0.008, 0.16)	0.08
GI^1^	−0.021	(−0.048, 0.006)	0.13	−0.025	(−0.054, 0.003)	0.08	−0.026	(−0.055, 0.004)	0.09
GL^1^	−0.001	(−0.004, 0.001)	0.27	−0.002	(−0.004, 0.001)	0.20	−0.001	(−0.004, 0.001)	0.28
II^1^	−0.002	(−0.009, 0.006)	0.63	−0.004	(−0.012, 0.004)	0.35	−0.004	(−0.012, 0.004)	0.29
IL^1^	−0.0003	(−0.0008, 0.0002)	0.23	−0.0003	(−0.0008, 0.0001)	0.17	−0.0003	(−0.0008, 0.0002)	0.24

B, unstandardized regression coefficient; CI, Confidence interval. *n* = 276 for all offspring; *n* = 135 for male offspring; *n* = 141 for female offspring. Model 1 = crude unadjusted model; Model 2 adjusts for: maternal age at recruitment, maternal pre-pregnancy BMI, length of gestation, offspring sex, parity and maternal height. Final model adjusted for: Model 2 plus smoking, physical activity, alcohol intake, energy intake (when not already considered in dietary score/%EI calculation), maternal education. E-DII, energy-adjusted Dietary Inflammatory Index; DASH, Dietary Approaches to Stop Hypertension; DAQ, Dietary Antioxidant Quality score; HEI, Healthy Eating Index; GI, glycaemic index; GL, glycaemic load; II, insulinemic index; IL, insulinemic load. Significant *p*-values are highlighted in bold.

1 Higher scores indicate more favorable dietary score.

2 Higher scores indicate less favorable dietary score.

#### 3.3.3. Maternal dietary scores and birth weight

The results for maternal diet associations with birth weight are shown in Table 5. Maternal E-DII and GI scores were positively associated with birth weight in the fully adjusted models (*p* < 0.05). Each unit increase in these dietary scores was associated with an increase in BW of 43.9 and 17.09 g respectively, indicating that a more pro-inflammatory diet or a diet that causes rapid increase in blood glucose levels is associated with higher birth weight.

**Table 5:** Associations between maternal dietary scores and birth weight (g) in the full sample and stratified by sex.

	Model 1 Unadjusted			Model 2			Model 3		
	B	95% CI	*p*-value	B	95% CI	*p*-value	B	95% CI	*p*-value
**All offspring**									
DII^1^	−6.23	(−42.29, 29.82)	0.73	29.91	(−14.48, 74.30)	0.19	41.37	(−3.80, 86.53)	0.07
E-DII^1^	15.35	(−22.75, 53.44)	0.43	31.90	(−5.66, 69.45)	0.10	43.90	(5.27, 82.53)	**0.03**
HEI-2015^2^	−0.54	(−8.13, 7.05)	0.89	−2.32	(−9.72, 5.08)	0.54	−4.43	(−12.10, 3.24)	0.26
DASH (frequencies)^2^	−4.08	(−16.85, 8.69)	0.53	−1.97	(−14.82, 10.88)	0.76	−7.22	(−20.93, 6.49)	0.30
DASH (weight)^2^	−5.08	(−17.30, 7.14)	0.41	−3.99	(−16.34, 8.37)	0.53	−10.10	(−23.51, 3.32)	0.14
DAQ General^2^	−20.73	(−61.86, 20.40)	0.32	−33.27	(−72.62, 6.08)	0.10	−37.45	(−76.92, 2.02)	0.06
DAQ Pregnancy^2^	−17.76	(−58.61, 23.10)	0.39	−30.67	(−69.83, 8.50)	0.12	−34.50	(−73.79, 4.79)	0.08
GI^1^	19.21	(5.93, 32.49)	**0.005**	14.03	(1.29, 26.78)	**0.03**	17.09	(3.97, 30.20)	**0.01**
GL^1^	0.66	(−0.58, 1.90)	0.30	0.59	(−0.56, 1.74)	0.31	0.60	(−0.59, 1.78)	0.32
II^1^	−2.34	(−6.22, 1.53)	0.23	−0.94	(−4.63, 2.75)	0.62	−0.99	(−4.73, 2.75)	0.60
IL^1^	0.25	(0.03, 0.47)	**0.03**	0.14	(−0.06, 0.35)	0.17	0.10	(−0.12, 0.31)	0.39
**Males**									
DII^1^	12.45	(−46.39, 71.30)	0.68	83.87	(15.07, 152.68)	**0.02**	97.74	(27.84, 167.64)	**0.01**
E-DII^1^	46.91	(−14.80, 108.62)	0.14	76.96	(19.21, 134.72)	**0.01**	92.20	(32.88, 151.52)	**0.003**
HEI-2015^2^	−6.76	(−18.33, 4.82)	0.25	−11.08	(−22.38, 0.22)	0.055	−14.09	(−25.91, −2.26)	**0.02**
DASH (frequencies)^2^	−10.40	(−31.82, 11.02)	0.34	−12.55	(−33.67, 8.57)	0.24	−21.61	(−45.07, 1.85)	0.07
DASH (weight)^2^	−11.81	(−32.33, 8.70)	0.26	−16.51	(−36.79, 3.77)	0.11	−27.37	(−49.91, −4.83)	**0.02**
DAQ General^2^	−19.34	(−86.36, 47.68)	0.57	−37.10	(−99.10, 24.90)	0.24	−43.01	(−105.79, 19.78)	0.18
DAQ Pregnancy^2^	−18.19	(−86.06, 49.67)	0.60	−36.83	(−99.38, 25.71)	0.25	−43.87	(−107.08, 19.33)	0.17
GI^1^	21.93	(1.40, 42.46)	**0.04**	19.78	(1.12, 38.44)	**0.04**	22.58	(3.24, 41.92)	**0.02**
GL^1^	0.95	(−1.11, 3.01)	0.36	1.09	(−0.74, 2.92)	0.24	1.05	(−0.87, 2.97)	0.28
II^1^	−2.02	(−8.38, 4.34)	0.53	0.48	(−5.26, 6.22)	0.87	0.33	(−5.45, 6.11)	0.91
IL^1^	0.22	(−0.11, 0.56)	0.19	0.14	(−0.16, 0.44)	0.35	0.08	(−0.24, 0.40)	0.62
**Females**									
DII^1^	−26.84	(−68.51, 14.83)	0.20	−23.91	(−78.40, 30.57)	0.39	−16.15	(−72.24, 39.95)	0.57
E-DII^1^	−20.72	(−65.00, 23.55)	0.36	−16.26	(−62.06, 29.55)	0.48	−9.24	(−56.90, 38.43)	0.70
HEI-2015^2^	5.86	(−2.74, 14.47)	0.18	8.76	(−0.53, 18.05)	0.06	8.42	(−1.08, 17.92)	0.08
DASH (frequencies)^2^	3.30	(−11.25, 17.86)	0.65	7.58	(−7.73, 22.90)	0.33	6.10	(−9.72, 21.93)	0.45
DASH (weight)^2^	2.61	(−11.31, 16.53)	0.71	7.11	(−7.53, 21.75)	0.34	5.02	(−10.49, 20.53)	0.52
DAQ General^2^	−20.65	(−68.65, 27.34)	0.40	−31.64	(−80.30, 17.02)	0.20	−24.69	(−73.96, 24.57)	0.32
DAQ Pregnancy^2^	−15.54	(−62.29, 31.20)	0.51	−25.82	(−73.30, 21.65)	0.28	−19.56	(−67.58, 28.46)	0.42
GI^1^	15.44	(−1.07, 31.95)	0.07	9.98	(−7.09, 27.05)	0.25	12.50	(−4.98, 29.98)	0.16
GL^1^	0.38	(−1.03, 1.80)	0.59	0.22	(−1.16, 1.61)	0.75	0.35	(−1.07, 1.77)	0.63
II^1^	−2.52	(−7.00, 1.97)	0.27	−1.99	(−6.62, 2.63)	0.40	−2.23	(−6.88, 2.42)	0.34
IL^1^	0.28	(−0.003, 0.57)	0.052	0.19	(−0.10, 0.47)	0.20	0.19	(−0.10, 0.49)	0.19

B = unstandardised regression coefficient, CI = Confidence interval. *n* = 276 for all offspring; *n* = 135 for male offspring; *n* = 141 for female offspring. Model 1 = crude unadjusted model; Model 2 adjusts for: maternal age at recruitment, maternal pre-pregnancy BMI, length of gestation, offspring sex, parity and maternal height. Final model adjusted for: Model 2 plus smoking, physical activity, alcohol intake, energy intake (when not already considered in dietary score/%EI calculation), maternal education. E-DII, energy-adjusted Dietary Inflammatory Index; DASH, Dietary Approaches to Stop Hypertension; DAQ, Dietary Antioxidant Quality score; HEI, Healthy Eating Index; GI, glycaemic index; GL, glycaemic load; II, insulinemic index; IL, insulinemic load.

1 Higher scores indicate more favorable dietary score. Significant *p*-values are highlighted in bold.

2 Higher scores indicate less favorable dietary score.

In sex-stratified analyses, the positive associations between the dietary inflammatory scores and birth weight in males persisted in the final model (B: 97.74, *p* = 0.007 and B: 92.20, *p* = 0.003 for the DII and E-DII scores respectively). A persistent positive association was also found between maternal GI and BW (B: 22.58, *p* = 0.02 in the fully adjusted model). Negative associations between HEI-2015 and DASH scores [based on intake amounts (grams/day)] with birth weight persisted in the fully adjusted models (B: −14.1, *p* = 0.02 and B: −27.4, *p* = 0.02 respectively). No significant associations were observed between any maternal dietary score and birth weight among female offspring. After correcting for multiple testing, the DII and E-DII score associations with birth weight in males remained statistically significant.

### 3.4. Sensitivity-to-outliers regression

Since the ordered leverage and Cook’s Distance values (*n* = 276) were less than 0.2 and less than 1 respectively, the 13 outliers were deemed as not highly influential points (48). Nonetheless, results of the sensitivity-to-outliers regression for glycemic and insulinemic scores are presented in Supplementary Tables 4–6.

Overall, the findings were minimally altered. For PW, the direction and magnitude of the initial GI associations were similar and were borderline significant in the fully adjusted model in the overall sample (B: 4.27, *p* = 0.051 vs. B: 4.13, *p* = 0.04 in main analysis) and for females (B: 6.02, *p* = 0.045 vs. B: 5.61, *p* = 0.04 in the main analysis). Excluding outliers showed maternal IL associations overall and in females (B: 0.124, *p* = 0.02 and B: 0.148, *p* = 0.03, respectively). Again, the effect change was similar to the original analysis (B: 0.06, *p* = 0.10 and B:0.07, *p* = 0.11, respectively).

Consistent with the results of the main analyses, no associations between maternal dietary scores and BW:PW ratio were found overall, except for negative associations between maternal GL and BW:PW ratio in female offspring in models 2 and 3 (B: −0.006, *p* = 0.03 and B: −0.005, *p* = 0.03). However, this finding did not withstand FDR correction. For BW, the overall positive GI association persisted and a similar direction and magnitude of effect was seen in males which was borderline significant (B: 19.53, *p* = 0.07 vs. B: 22.58, *p* = 0.02 in the main fully adjusted model).

### 3.5. Sensitivity analyses

Results from sensitivity analyses examining dietary-placental associations restricted to term births (*n* = 248) are shown in Supplementary Tables 1–3. There were 12 infants with missing gestational age data, five infants born post-term and 11 preterm births that were excluded from the sensitivity analyses. Results suggest attenuated associations for some maternal dietary scores with outcomes, but also new associations in the fully adjusted model. The negative associations between both DAQ scores and the HEI-2015 with PW remained unchanged as well as the positive association between maternal GI and PW (Supplementary Table 1). However, the previously observed positive association between PW and E-DII was attenuated. Interestingly, a new significant positive association was observed for the DASH score (based on food amounts). A higher DASH score indicative of higher maternal diet quality was associated with lower placental weights in term infants (B: −4.38, *p* = 0.04 vs. B: −2.23, *p* = 0.22 in the original analysis). A new positive association was found between the HEI-2015 score and BW:PW ratio in the fully adjusted model (B:0.015, *p* = 0.01 compared to original analysis results of B: 0.011, *p* = 0.07), suggesting that higher diet quality is associated with improved placental efficiency in term infants (Supplementary Table 2). For birth weight, the positive association remained for maternal GI in the sensitivity analyses but not for the E-DII score (Supplementary Table 3). Interestingly, the general DAQ score and the DAQ pregnancy-specific score were negatively associated with birth weight when restricting to term births, suggesting that better dietary antioxidant status is associated with lower birth weights in term infants (B: −41.918, *p* = 0.04 and B: −39.633, *p* = 0.05 respectively, compared to original analysis results of B: −37.45, *p* = 0.06 and B: −34.50, *p* = 0.09).

When stratifying by sex, restricting to term births did not largely alter placental weight, BW:PW, or BW findings (Supplementary Tables 1–3) and were of a similar magnitude to the original analyses. There were some attenuations in the associations with DII in males and pregnancy-specific DAQ in females (Supplementary Table 1), with a new BW association appearing for males only with the DASH (based on frequencies) score (Supplementary Table 3).

## 4. Discussion

Dietary-placental weight associations were observed with the E-DII, HEI-2015, GI, and DAQ scores. The DAQ and HEI-2015 negative associations were robust to adjustment for a comprehensive list of *a priori* covariates and in sensitivity analyses restricted to term births, highlighting the potential importance of antioxidant status and diet quality in placental growth and development. When stratified by sex, maternal GI was associated with increased PW in female offspring, whereas a more pro-inflammatory (higher E-DII) and low quality (lower HEI-2015) diet were associated with higher PW in males, suggesting evidence of sexual dimorphism.

### 4.1. Dietary status and placental outcomes

PW has been negatively correlated with a maternal fast food-like dietary pattern (49) while another study found low PW in mothers with low adherence to a Mediterranean-like dietary pattern (50). Here, higher diet quality (based on the HEI-2015 score) was associated with lower PW. No other studies have investigated HEI associations with PW. However, a recent study found that averaged HEI-2010 scores during pregnancy were associated with the global placental metabolome, suggesting that maternal diet quality may affect the metabolism of fatty acids and branch chain amino acids which could lead to changes in nutrient delivery to the offspring *via* the placenta (51). Recent evidence from the Generation R study (*n* = 3,414) suggests that improving maternal adherence to the DASH diet leads to improved fetoplacental vascular function, which may be explained by improved endothelial cell function and reduction of oxidative stress (52). However, we did not find significant placental associations with DASH scores and this could be due to our smaller sample size and our different outcome measurement of placental weight rather than placental hemodynamics (using ultrasound methods) used in their study.

Animal studies suggest that oxidative stress or inflammation caused by a pro-inflammatory, high-fat diet results in decreased microvessel density and number of placental trophoblasts (53). In our analysis, the maternal E-DII score was positively associated with PW. This is in contrast to findings from secondary analysis of the PEARS RCT (*n* = 434) which showed no association between the E-DII score and PW (20). It should be noted that the PEARS trial comprised women with overweight and obesity, which represent only 24% of the Lifeways subsample, indicating potentially distinct placental inflammatory pathways depending on maternal BMI as previously suggested by Aye et al. (54).

Higher DAQ scores were associated with higher BW:PW ratios in the crude models of our study which supports findings in sheep of improved antioxidant status and increased placental efficiency after vitamin C and E supplementation (55). Examination of the DAQ score with placental outcomes in humans is non-existent, but observational research suggests that oxidative stress or inflammation caused by a pro-inflammatory diet results in higher resistance and lower flow placental circulation (56).

It is thought that maternal hyperglycemia and hyperinsulinemia contributes to changes in placental growth and vasculature (57). While we did not observe any associations of maternal insulinemic indices with offspring outcomes, there was a positive association between maternal GI and PW in our study. No further associations between maternal GI and BW:PW ratio were detected, nor were any associations found between maternal GL and PW or with BW:PW ratio in our study, which is similar to null findings from previous work on the full Lifeways cohort (*n* = 842) which examined maternal GI and GL associations with birth outcomes (43).

### 4.2. Sexual dimorphism

While we did not observe differences in mean placental weights between male and female offspring, we report some evidence (without formal tests) that maternal dietary associations with placental outcomes might be driven more by one sex than the other. In the current analysis, lower dietary inflammation and higher diet quality were associated with lower PW, mainly driven by male offspring. While not specific to PW, our findings support those from the larger pooled ALPHABET analysis, where E-DII and DASH associations with birth weight and risk of SGA were stronger in the male offspring (8). In a recent subsample of mothers from the Healthy Start US cohort (*n* = 108), higher maternal HEI was associated with lower abundance of placental p38MAPK proteins in males (58). The p38MAPK cascade is a major signaling cascade linking proinflammatory cytokines, oxidative stress, and other physiological stress to apoptosis, and possibly cell growth and placental angiogenesis. Hence, maternal diet quality could have a role in mitigating this cascade effect *via* changes in placental gene expression, particularly in male offspring.

Additionally, we report that a higher pregnancy-specific DAQ score was associated with lower PW in females only. Placental adaptations and growth strategies vary in a sex-specific manner (59), with a greater vulnerability of males for placental disorders (60). A small human study (*n* = 56) observed higher total antioxidant status and lower plasma membrane hydroperoxides in the umbilical cord artery of females compared with the males (61). Research has suggested a protective effect of estradiol in females on decreasing free radical production *via* upregulation of antioxidant genes (62), but future work is needed to explore the effect of antioxidants on placental morphology.

Due to the small magnitude of our glycemic score findings in this stratified analysis, results should be interpreted with caution when it comes to fetal programming. Because there is an enhanced presence of placental insulin receptors on the maternal villous membrane early in gestation (63), timing of exposure to maternal diet should be considered when exploring the potentially greater effect of a high-sugar diet on the metabolic phenotype in female offspring compared to males, which is thought to be mediated *via* changes in placental structure and gene expression (15).

### 4.3. Sensitivity analyses

In the sensitivity analyses restricted to term births, negative associations between the HEI-2015 and the DAQ scores with PW persisted, suggesting robustness of our overall findings that higher maternal diet quality and better antioxidant status are associated with lower PW. The overall E-DII and GI associations were attenuated, highlighting the importance of investigating these dietary-placental associations in higher-risk groups such as mothers with glucose intolerance, obesity and macrosomia who are more likely to have pre-term delivery (64, 65). Sex-specific results were also robust and main conclusions remained unchanged for placental weight associations with E-DII and HEI-2015 scores for males; and PW associations with maternal GI in female offspring.

Briefly, the HEI-2015, DASH, DII, E-DII, and GI birth weight associations found in male offspring restricted to term births also suggest the greater sensitivity of males to maternal diet and adverse maternal nutritional environments (i.e., marked by pro-inflammatory and low-quality diets) that have been proposed in the literature (11, 15, 17).

### 4.4. Strengths and limitations

Strengths of this study include the use of a validated FFQ and a variety of validated dietary scores in our study which is also a well-characterized cohort that is representative of the general obstetric Irish population. While we have controlled for several potential confounders, there may be unmeasured confounders. Maternal pre-pregnancy weight and BMI were self-reported and the use of FFQ may also under- or over-estimate the food intake of respondents. Systematic error arising from self-reported FFQs was reduced through exclusion of mothers with implausible energy intakes and energy adjustment for dietary scores (31). While the standard approach has been to measure trimmed PW, untrimmed PW was recorded and examined in this study. Reassuringly however, Leary et al. (66) have suggested that trimmed and untrimmed PW are exchangeable, based on their high correlation. Correction for multiple testing using the Benjamini-Hochberg procedure was also conducted. This method provides the right balance between discovering statistically significant effects and limitations by false positives which is most appropriate for the current exploratory statistical analysis (67).

## 5. Conclusion

In conclusion, our analysis showed that in an Irish general pregnant population, maternal dietary indices including the E-DII, GI, HEI-2015, and DAQ scores were associated with placental weight. The placenta may be an important mediator of sexual dimorphism in fetal development programming and in offspring outcomes. Our findings suggest that female offspring may be more sensitive to increased glucose levels while male offspring by *in-utero* stresses that are regulated by inflammatory pathways and overall diet quality. Since diet is only one aspect of overall lifestyle, future research including other maternal lifestyle behaviors (e.g., sleep and exercise as well as diet) in longitudinal and interventional studies, of larger sample sizes and that include placental parameters as their main outcome of interest, are warranted.

## Data availability statement

The data that support the findings of this study are available from the Lifeways Cross-Generational Cohort Study but restrictions apply to the availability of these data, which were used under license for the current study, and so are not publicly available. Data are however available from the authors upon reasonable request and with permission from the study executive committee.

## Ethics statement

The studies involving human participants were reviewed and approved by Ethic Committees of the Coombe University Hospital, Dublin, University College Dublin, Irish College of General Practitioners and University College Hospital, Galway, Ireland. The patients/participants provided their written informed consent to participate in this study.

## Author contributions

ST and CP: conceptualization, visualization, and writing—review and editing. ST, CM, JM, JH, CK, and CP: methodology. CM and CK: investigation. JM and AD: data curation. ST and RS: formal analysis. JM, AD, and JH: software. JM, CK and CP: resources. CK and CP: project administration and funding acquisition. CP: supervision and validation. ST: writing—original draft preparation. All authors contributed to the article and approved the submitted version.

## Funding

Work on the Lifeways Cross-Generation Cohort Study was funded by the Health Research Board, Ireland (reference HRC/2007/13) and is overseen by an inter-disciplinary steering group. The current research is supported by an Ad Astra Fellowship (grant number R20719) and Ad Astra Studentship (grant number R20720) from University College Dublin.

## Conflict of interest

We wish to disclose that JH owns controlling interest in Connecting Health Innovations LLC (CHI), a company that has licensed the right to his invention of the dietary inflammatory index (DII) from the University of South Carolina to develop computer and smartphone applications for patient counseling and dietary intervention in clinical settings. The subject matter of this paper will not have any direct bearing on that work, nor has that activity exerted any influence on this project.The remaining authors declare that the research was conducted in the absence of any commercial or financial relationships that could be construed as a potential conflict of interest.

## Publisher’s note

All claims expressed in this article are solely those of the authors and do not necessarily represent those of their affiliated organizations, or those of the publisher, the editors and the reviewers. Any product that may be evaluated in this article, or claim that may be made by its manufacturer, is not guaranteed or endorsed by the publisher.
