# Supplementary material for: Associations between maternal dietary scores during early pregnancy with placental outcomes
Source: Front Nutr. 2023 Feb 8;10:1060709. doi: 10.3389/fnut.2023.1060709 (PMC9945217; doi:10.3389/fnut.2023.1060709)
Supplement: Supplementary file 1 [file Data_Sheet_1.docx]

Supplementary Material

# Supplementary Figures and Tables

**Supplementary Figure 1** Lifeways participant flow chart

1138 children born to 1126 recruited mothers

**Supplementary Table 1: Sensitivity Analysis of the associations between maternal dietary scores and placental weight (g), excluding pre- and post-term births**

Miscarriages/Stillbirth (*n*= 23)

Maternal death (*n*= 1)

Withdrawal before birth (*n*= 2)

Missing hospital records (*n*= 18)

Other (n=11)

Twins (n= 24)

Singleton live births (n= 1114)

Available families at birth (*n*= 1059)

Singleton mothers with FFQ data (*n*= 1051)

Plausible intake maternal data (n=938)

**Galway participants with maternal dietary data and placental weights:**

DASH/DII/E-DII/HEI (n= 276)

DAQ Scores (n=274)

GI/GL/II/IL (n=263)

| **All Offspring** |  | **B** | **95% CI** | **p-value** |
| --- | --- | --- | --- | --- |
|  |  |  |  |  |
| DII^1^ | n=248 | 9.62 | (-4.56, 23.80) | 0.18 |
| E-DII^1^ |  | 10.73 | (-1.34, 22.81) | 0.08 |
| HEI-2015^2^ |  | -3.40 | (-5.77, -1.04) | **0.005** |
| DASH (frequencies) ^2^ |  | -4.20 | (-8.46, 0.07) | 0.054 |
| DASH (weight) ^2^ |  | -4.38 | (-8.54, -0.21) | **0.04** |
| DAQ General^2^ | n=246 | -15.59 | (-28.18, -3.00) | **0.02** |
| DAQ Pregnancy^2^ |  | -14.58 | (-26.96, -2.19) | **0.02** |
| GI^1^ | n=248 | 4.19 | (-0.16, 8.54) | 0.06 |
| GL^1^ |  | 0.23 | (-0.16, 0.61) | 0.24 |
| II^1^ |  | -0.41 | (-1.61, 0.80) | 0.51 |
| IL^1^ |  | 0.05 | (-0.02, 0.12) | 0.16 |
|  |  |  |  |  |
| **Males** |  |  |  |  |
|  |  | **B** | **95% CI** | **p-value** |
| DII^1^ | n=117 | 22.56 | (-1.43, 46.54) | 0.06 |
| E-DII^1^ |  | 23.46 | (3.26, 43.67) | **0.02** |
| HEI-2015^2^ |  | -5.30 | (-9.20, -1.41) | **0.01** |
| DASH (frequencies) ^2^ |  | -5.37 | (-13.25, 2.51) | 0.18 |
| DASH (weight) ^2^ |  | -6.57 | (-14.05, 0.90) | 0.08 |
| DAQ General^2^ | n=116 | -15.49 | (-36.69, 5.71) | 0.15 |
| DAQ Pregnancy^2^ |  | -14.10 | (-35.25, 7.04) | 0.19 |
| GI^1^ | n=117 | 2.72 | (-4.44, 9.89) | 0.45 |
| GL^1^ |  | 0.20 | (-0.46, 0.86) | 0.54 |
| II^1^ |  | -0.86 | (-2.82, 1.10) | 0.39 |
| IL^1^ |  | 0.04 | (-0.07, 0.15) | 0.46 |
|  |  |  |  |  |
| **Females** |  |  |  |  |
|  |  | **B** | **95% CI** | **p-value** |
| DII^1^ | n=131 | -4.62 | (-21.63, 12.39) | 0.59 |
| E-DII^1^ |  | -4.25 | (-18.62, 10.12) | 0.56 |
| HEI-2015^2^ |  | -0.65 | (-3.61, 2.31) | 0.66 |
| DASH (frequencies) ^2^ |  | -2.23 | (-7.07, 2.61) | 0.36 |
| DASH (weight) ^2^ |  | -1.71 | (-6.47, 3.04) | 0.48 |
| DAQ General^2^ | n=130 | -12.20 | (-27.54, 3.15) | 0.12 |
| DAQ Pregnancy^2^ |  | -12.48 | (-27.23, 2.27) | 0.10 |
| GI^1^ | n=131 | 6.37 | (1.02, 11.73) | **0.02** |
| GL^1^ |  | 0.24 | (-0.22, 0.70) | 0.31 |
| II^1^ |  | 0.27 | (-1.20, 1.74) | 0.71 |
| IL^1^ |  | 0.07 | (-0.03, 0.16) | 0.16 |

B= unstandardised regression coefficient, CI = Confidence interval

Multivariable model adjusted for maternal age at recruitment, maternal pre-pregnancy BMI, length of gestation, offspring gender, parity, maternal energy intake (except for dietary scores where energy has already been intrinsically adjusted), maternal education, smoking, alcohol intake and physical activity.

^1^Higher scores indicate more favourable dietary score

^2^Higher scores indicate less favourable dietary score

**Supplementary Table 2: Sensitivity Analysis associations between maternal dietary scores and Birth weight: Placental Weight (BW:PW) Ratio, excluding pre- and post-term births**

| **All Offspring** |  | **B** | **95% CI** | **p-value** |
| --- | --- | --- | --- | --- |
|  |  |  |  |  |
| DII^1^ | n=248 | -0.03 | (-0.09, 0.04) | 0.47 |
| E-DII^1^ |  | -0.03 | (-0.09, 0.03) | 0.36 |
| HEI-2015^2^ |  | 0.01 | (0.003, 0.03) | **0.01** |
| DASH (frequencies) ^2^ |  | 0.02 | (-0.01, 0.04) | 0.15 |
| DASH (weight) ^2^ |  | 0.01 | (-0.01, 0.03) | 0.17 |
| DAQ General^2^ | n=246 | 0.05 | (-0.01, 0.11) | 0.10 |
| DAQ Pregnancy^2^ |  | 0.05 | (-0.01, 0.11) | 0.13 |
| GI^1^ | n=248 | -0.01 | (-0.03, 0.02) | 0.60 |
| GL^1^ |  | -0.001 | (-0.003, 0.001) | 0.42 |
| II^1^ |  | 0.0003 | (-0.005, 0.006) | 0.93 |
| IL^1^ |  | -0.0001 | (-0.0004, 0.0002) | 0.55 |
|  |  |  |  |  |
| **Males** |  |  |  |  |
|  |  | **B** | **95% CI** | **p-value** |
| DII^1^ | n=117 | -0.04 | (-0.14, 0.07) | 0.47 |
| E-DII^1^ |  | -0.04 | (-0.13, 0.05) | 0.38 |
| HEI-2015^2^ |  | 0.01 | (-0.003, 0.03) | 0.10 |
| DASH (frequencies) ^2^ |  | 0.0005 | (-0.03, 0.03) | 0.98 |
| DASH (weight) ^2^ |  | 0.003 | (-0.03, 0.03) | 0.87 |
| DAQ General^2^ | n=116 | 0.04 | (-0.06, 0.13) | 0.44 |
| DAQ Pregnancy^2^ |  | 0.02 | (-0.07, 0.11) | 0.60 |
| GI^1^ | n=117 | 0.014 | (-0.02, 0.04) | 0.36 |
| GL^1^ |  | 0.0001 | (-0.003, 0.003) | 0.93 |
| II^1^ |  | 0.003 | (-0.005, 0.011) | 0.45 |
| IL^1^ |  | -0.000014 | (-0.0005, 0.0004) | 0.95 |
|  |  |  |  |  |
| **Females** |  |  |  |  |
|  |  | **B** | **95% CI** | **p-value** |
| DII^1^ | n=131 | 0.01 | (-0.08, 0.10) | 0.83 |
| E-DII^1^ |  | 0.01 | (-0.07, 0.09) | 0.78 |
| HEI-2015^2^ |  | 0.01 | (-0.005, 0.03) | 0.17 |
| DASH (frequencies) ^2^ |  | 0.02 | (-0.01, 0.05) | 0.13 |
| DASH (weight) ^2^ |  | 0.02 | (-0.01, 0.04) | 0.21 |
| DAQ General^2^ | n=130 | 0.06 | (-0.03, 0.14) | 0.18 |
| DAQ Pregnancy^2^ |  | 0.06 | (-0.02, 0.14) | 0.11 |
| GI^1^ | n=131 | -0.03 | (-0.056, 0.003) | 0.08 |
| GL^1^ |  | -0.001 | (-0.004, 0.001) | 0.34 |
| II^1^ |  | -0.004 | (-0.012, 0.004) | 0.35 |
| IL^1^ |  | -0.0002 | (-0.0007, 0.0003) | 0.44 |

B= unstandardised regression coefficient, CI = Confidence interval

Multivariable model adjusted for maternal age at recruitment, maternal pre-pregnancy BMI, length of gestation, offspring gender, parity, maternal energy intake (except for dietary scores where energy has already been intrinsically adjusted), maternal education, smoking, alcohol intake and physical activity.

^1^Higher scores indicate more favourable dietary score

^2^Higher scores indicate less favourable dietary score

**Supplementary Table 3: Sensitivity Analysis associations between maternal dietary scores and birth weight (g), excluding pre- and post-term births**

| **All Offspring** |  | **B** | **95% CI** | **p-value** |
| --- | --- | --- | --- | --- |
|  |  |  |  |  |
| DII^1^ | n=248 | 30.70 | (-13.83, 75.22) | 0.18 |
| E-DII^1^ |  | 33.37 | (-4.63, 71.37) | 0.08 |
| HEI-2015^2^ |  | -4.69 | (-12.23, 2.84) | 0.22 |
| DASH (frequencies) ^2^ |  | -9.63 | (-23.10, 3.85) | 0.16 |
| DASH (weight) ^2^ |  | -10.02 | (-23.19, 3.14) | 0.13 |
| DAQ General^2^ | n=246 | -41.92 | (-81.60, -2.24) | **0.04** |
| DAQ Pregnancy^2^ |  | -39.63 | (-78.64, -0.62) | **0.046** |
| GI^1^ | n=248 | 18.05 | (4.37, 31.72) | **0.01** |
| GL^1^ |  | 0.90 | (-0.32, 2.11) | 0.15 |
| II^1^ |  | -1.06 | (-4.87, 2.76) | 0.59 |
| IL^1^ |  | 0.18 | (-0.04, 0.40) | 0.12 |
|  |  |  |  |  |
| **Males** |  |  |  |  |
|  |  | **B** | **95% CI** | **p-value** |
| DII^1^ | n=117 | 87.26 | (14.63, 159.89) | **0.02** |
| E-DII^1^ |  | 84.96 | (23.45, 146.47) | **0.01** |
| HEI-2015^2^ |  | -15.42 | (-27.38, -3.46) | **0.01** |
| DASH (frequencies) ^2^ |  | -25.17 | (-49.11, -1.22) | **0.04** |
| DASH (weight) ^2^ |  | -26.64 | (-49.37, -3.91) | **0.02** |
| DAQ General^2^ | n=116 | -51.19 | (-115.98, 13.59) | 0.12 |
| DAQ Pregnancy^2^ |  | -51.60 | (-116.07, 12.88) | 0.12 |
| GI^1^ | n=117 | 24.32 | (2.75, 45.88) | **0.03** |
| GL^1^ |  | 1.42 | (-0.59, 3.42) | 0.16 |
| II^1^ |  | -0.23 | (-6.28, 5.82) | 0.94 |
| IL^1^ |  | 0.17 | (-0.17, 0.50) | 0.32 |
|  |  |  |  |  |
| **Females** |  |  |  |  |
|  |  | **B** | **95% CI** | **p-value** |
| DII^1^ | n=131 | -16.70 | (-72.70, 39.30) | 0.56 |
| E-DII^1^ |  | -11.89 | (-59.26, 35.48) | 0.62 |
| HEI-2015^2^ |  | 7.02 | (-2.66, 16.70) | 0.15 |
| DASH (frequencies) ^2^ |  | 2.50 | (-13.49, 18.49) | 0.76 |
| DASH (weight) ^2^ |  | 2.53 | (-13.17, 18.22) | 0.75 |
| DAQ General^2^ | n=130 | -25.85 | (-76.70, 25.01) | 0.32 |
| DAQ Pregnancy^2^ |  | -21.81 | (-70.81, 27.19) | 0.38 |
| GI^1^ | n=131 | 14.39 | (-3.47, 32.25) | 0.11 |
| GL^1^ |  | 0.57 | (-0.94, 2.09) | 0.46 |
| II^1^ |  | -1.80 | (-6.64, 3.04) | 0.46 |
| IL^1^ |  | 0.23 | (-0.08, 0.54) | 0.15 |

B = unstandardised regression coefficient, CI = Confidence interval

Multivariable model adjusted for maternal age at recruitment, maternal pre-pregnancy BMI, length of gestation, offspring gender, parity, maternal energy intake (except for dietary scores where energy has already been intrinsically adjusted), maternal education, smoking, alcohol intake and physical activity.

^1^Higher scores indicate more favourable dietary score

^2^Higher scores indicate less favourable dietary score

**Supplementary Table 4: Associations between maternal glycemic and insulinemic scores with placental weight (g) in the full sample and stratified by sex, outliers removed**

| **All Offspring** | | **Model 1 Unadjusted** | | | **Model 2** | | | **Model 3** | | |
| --- | --- | --- | --- | --- | --- | --- | --- | --- | --- | --- |
|  |  | B | 95% CI | p-value | B | 95% CI | p-value | B | 95% CI | p-value |
| GI | n=263 | 4.83 | (1.01, 8.64) | **0.01** | 3.38 | (-0.85, 7.61) | 0.12 | 4.27 | (-0.03, 8.57) | 0.05 |
| GL |  | 0.34 | (0.00, 0.69) | 0.05 | 0.43 | (-0.21, 1.07) | 0.19 | 0.43 | (-0.22, 1.08) | 0.20 |
| II |  | 0.04 | (-1.41, 1.48) | 0.96 | 0.03 | (-1.52, 1.59) | 0.97 | 0.04 | (-1.54, 1.62) | 0.96 |
| IL |  | 0.11 | (0.04, 0.18) | **0.00** | 0.14 | (0.04, 0.24) | **0.01** | 0.12 | (0.02, 0.23) | **0.02** |
|  |  |  |  |  |  |  |  |  |  |  |
|  |  |  |  |  |  |  |  |  |  |  |
| Males |  |  |  |  |  |  |  |  |  |  |
| GI | n=127 | 3.58 | (-2.28, 9.43) | 0.23 | 2.32 | (-3.95, 8.59) | 0.46 | 3.37 | (-2.99, 9.74) | 0.30 |
| GL |  | 0.42 | (-0.13, 0.98) | 0.13 | 0.30 | (-0.65, 1.25) | 0.53 | 0.28 | (-0.71, 1.27) | 0.57 |
| II |  | -0.52 | (-2.99, 1.95) | 0.68 | 0.12 | (-2.44, 2.67) | 0.93 | 0.05 | (-2.56, 2.65) | 0.97 |
| IL |  | 0.13 | (0.03, 0.24) | **0.01** | 0.16 | (0.004, 0.32) | **0.05** | 0.13 | (-0.05, 0.30) | 0.15 |
|  |  |  |  |  |  |  |  |  |  |  |
|  |  |  |  |  |  |  |  |  |  |  |
| Females |  |  |  |  |  |  |  |  |  |  |
| GI | n=136 | 6.44 | (1.54, 11.34) | **0.01** | 5.39 | (-0.28, 11.07) | 0.06 | 6.02 | (0.13, 11.91) | **0.05** |
| GL |  | 0.26 | (-0.16, 0.68) | 0.22 | 0.81 | (-0.03, 1.66) | 0.06 | 0.82 | (-0.06, 1.69) | 0.07 |
| II |  | 0.52 | (-1.16, 2.19) | 0.54 | 0.68 | (-1.22, 2.58) | 0.48 | 0.65 | (-1.29, 2.59) | 0.51 |
| IL |  | 0.09 | (-0.001, 0.17) | 0.05 | 0.15 | (0.02, 0.27) | **0.02** | 0.15 | (0.02, 0.28) | **0.03** |

B = unstandardised regression coefficient, CI = Confidence interval

Model 1=crude unadjusted model; Model 2 adjusts for: maternal age at recruitment, maternal pre-pregnancy BMI, length of gestation, offspring sex, parity and maternal height; Final model adjusted for: Model 2 plus smoking, physical activity, alcohol intake, energy intake (when not already considered in dietary score/%EI calculation), maternal education

GI, glycaemic index; GL, glycaemic load; II: insulinemic index; IL: insulinemic load**Supplementary Table 5: Associations between maternal glycemic and insulinemic scores with BW:PW ratio in the full sample and stratified by sex, outliers removed.**

| **All Offspring** | | **Model 1 Unadjusted** | | | **Model 2** | | | **Model 3** | | |
| --- | --- | --- | --- | --- | --- | --- | --- | --- | --- | --- |
|  |  | B | 95% CI | p-value | B | 95% CI | p-value | B | 95% CI | p-value |
| GI | n=263 | -0.008 | (-0.027, 0.011) | 0.40 | -0.009 | (-0.030, 0.013) | 0.43 | -0.010 | (-0.031, 0.012) | 0.39 |
| GL |  | -0.001 | (-0.003, 0.000) | 0.08 | -0.002 | (-0.006, 0.001) | 0.15 | -0.002 | (-0.006, 0.001) | 0.16 |
| II |  | -0.003 | (-0.010, 0.004) | 0.35 | -0.003 | (-0.011, 0.005) | 0.41 | -0.004 | (-0.012, 0.004) | 0.34 |
| IL |  | 0.000 | (-0.001, 0.000) | 0.08 | 0.000 | (-0.001, 0.000) | 0.08 | 0.000 | (-0.001, 0.000) | 0.08 |
|  |  |  |  |  |  |  |  |  |  |  |
|  |  |  |  |  |  |  |  |  |  |  |
| Males |  |  |  |  |  |  |  |  |  |  |
| GI | n=127 | 0.003 | (-0.02, 0.03) | 0.82 | 0.007 | (-0.02, 0.04) | 0.65 | 0.005 | (-0.02, 0.03) | 0.73 |
| GL |  | -0.001 | (-0.004, 0.001) | 0.22 | -0.001 | (-0.005, 0.003) | 0.70 | -0.001 | (-0.005, 0.004) | 0.75 |
| II |  | -0.002 | (-0.01, 0.009) | 0.73 | -0.001 | (-0.012, 0.01) | 0.89 | 0.000 | (-0.012, 0.012) | 0.96 |
| IL |  | 0.000 | (-0.001, 0.000) | 0.17 | 0.000 | (-0.001, 0.00) | 0.32 | 0.000 | (-0.001, 0.00) | 0.36 |
|  |  |  |  |  |  |  |  |  |  |  |
|  |  |  |  |  |  |  |  |  |  |  |
| Females |  |  |  |  |  |  |  |  |  |  |
| GI | n=135 | -0.022 | (-0.05, 0.01) | 0.12 | -0.026 | (-0.06, 0.007) | 0.12 | -0.028 | (-0.06, 0.006) | 0.11 |
| GL |  | -0.001 | (-0.004, 0.001) | 0.23 | -0.006 | (-0.010, -0.001) | **0.03** | -0.005 | (-0.01, -0.001) | **0.03** |
| II |  | -0.005 | (-0.014, 0.005) | 0.35 | -0.008 | (-0.02, 0.003) | 0.17 | -0.008 | (-0.019, 0.003) | 0.13 |
| IL |  | 0.000 | (-0.001, 0.00) | 0.26 | -0.001 | (-0.001, 0.00) | 0.11 | -0.001 | (-0.001, 0.00) | 0.13 |

B = unstandardised regression coefficient, CI = Confidence interval

Model 1=crude unadjusted model; Model 2 adjusts for: maternal age at recruitment, maternal pre-pregnancy BMI, length of gestation, offspring sex, parity and maternal height; Final model adjusted for: Model 2 plus smoking, physical activity, alcohol intake, energy intake (when not already considered in dietary score/%EI calculation), maternal education

GI, glycaemic index; GL, glycaemic load; II: insulinemic index; IL: insulinemic load

**Supplementary Table 6: Associations between maternal glycemic and insulinemic scores with birthweight (g) in the full sample and stratified by sex, outliers removed.**

| **All Offspring** | | **Model 1 Unadjusted** | | | **Model 2** | | | **Model 3** | | |
| --- | --- | --- | --- | --- | --- | --- | --- | --- | --- | --- |
|  |  | B | 95% CI | p-value | B | 95% CI | p-value | B | 95% CI | p-value |
| GI | n=263 | 18.94 | (4.89, 33.00) | **0.01** | 11.53 | (-2.73, 25.78) | 0.11 | 15.35 | (0.96, 29.73) | **0.04** |
| GL |  | 0.74 | (-0.54, 2.02) | 0.26 | 0.47 | (-1.69, 2.63) | 0.67 | 0.45 | (-1.73, 2.64) | 0.68 |
| II |  | -2.60 | (-7.93, 2.73) | 0.34 | -2.60 | (-7.84, 2.63) | 0.33 | -2.87 | (-8.13, 2.40) | 0.28 |
| IL |  | 0.31 | (0.05, 0.56) | **0.02** | 0.31 | (-0.03, 0.65) | 0.07 | 0.20 | (-0.15, 0.56) | 0.26 |
|  |  |  |  |  |  |  |  |  |  |  |
|  |  |  |  |  |  |  |  |  |  |  |
| Males |  |  |  |  |  |  |  |  |  |  |
| GI | n=127 | 19.08 | (-3.03, 41.18) | 0.09 | 15.69 | (-5.10, 36.48) | 0.14 | 19.53 | (-1.27, 40.32) | 0.07 |
| GL |  | 0.91 | (-1.22, 3.04) | 0.40 | 0.60 | (-2.59, 3.78) | 0.71 | 0.57 | (-2.69, 3.84) | 0.73 |
| II |  | -4.35 | (-13.71, 5.01) | 0.36 | -0.72 | (-9.26, 7.82) | 0.87 | -0.74 | (-9.36, 7.87) | 0.86 |
| IL |  | 0.34 | (-0.06, 0.75) | 0.10 | 0.36 | (-0.17, 0.89) | 0.18 | 0.21 | (-0.36, 0.78) | 0.47 |
|  |  |  |  |  |  |  |  |  |  |  |
| Females |  |  |  |  |  |  |  |  |  |  |
|  |  |  |  |  |  |  |  |  |  |  |
| GI | n=135 | 18.62 | (1.45, 35.79) | **0.03** | 11.11 | (-8.06, 30.28) | 0.25 | 14.08 | (-5.39, 33.56) | 0.15 |
| GL |  | 0.59 | (-0.87, 2.06) | 0.43 | 0.48 | (-2.39, 3.36) | 0.74 | 0.54 | (-2.36, 3.44) | 0.71 |
| II |  | -1.03 | (-6.85, 4.80) | 0.73 | -2.57 | (-8.93, 3.79) | 0.43 | -2.93 | (-9.29, 3.42) | 0.36 |
| IL |  | 0.27 | (-0.03, 0.57) | 0.08 | 0.32 | (-0.10, 0.75) | 0.13 | 0.30 | (-0.14, 0.74) | 0.18 |

B = unstandardised regression coefficient, CI = Confidence interval

Model 1=crude unadjusted model; Model 2 adjusts for: maternal age at recruitment, maternal pre-pregnancy BMI, length of gestation, offspring sex, parity and maternal height; Final model adjusted for: Model 2 plus smoking, physical activity, alcohol intake, energy intake (when not already considered in dietary score/%EI calculation), maternal education

GI, glycaemic index; GL, glycaemic load; II: insulinemic index; IL: insulinemic load

**
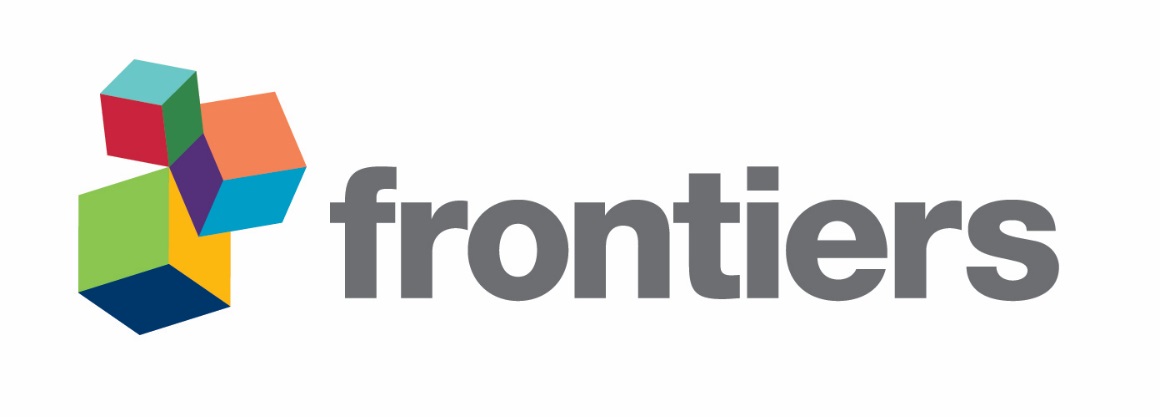
**
