# Supplementary material for: Associations between maternal dietary scores during early pregnancy with placental outcomes
Source: Front Nutr. 2023 Feb 8;10:1060709. doi: 10.3389/fnut.2023.1060709 (PMC9945217; doi:10.3389/fnut.2023.1060709)

Supplementary Material

1 Supplementary Figures and Tables

Supplementary Figure 1. Lifeways participant flow chart

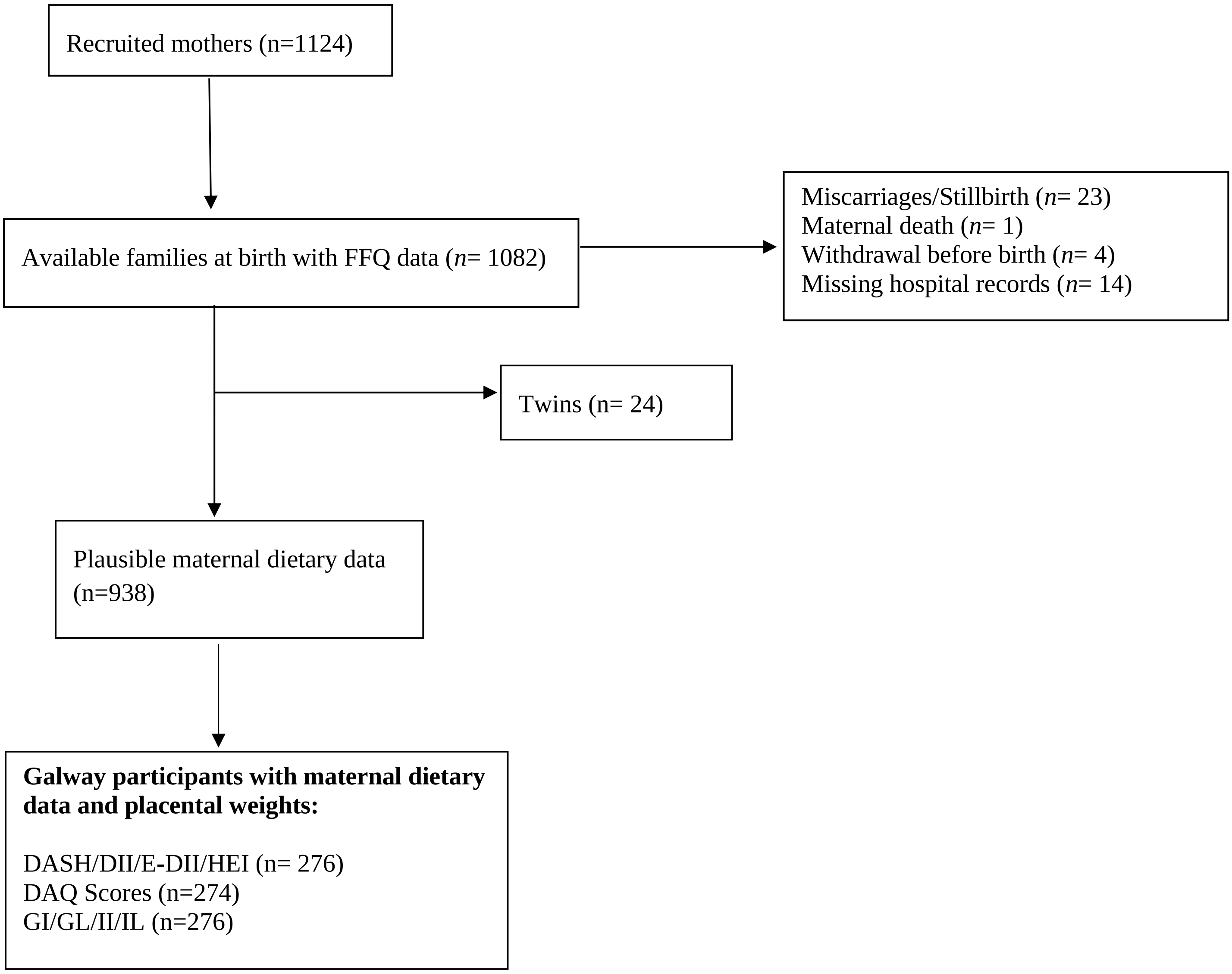

Supplement: Supplementary file 2 [file Image_1.pdf]
